# A review of typhoid fever transmission dynamic models and economic evaluations of vaccination

**DOI:** 10.1016/j.vaccine.2015.04.013

**Published:** 2015-06-19

**Authors:** Conall H. Watson, W. John Edmunds

**Affiliations:** Centre for the Mathematical Modelling of Infectious Diseases, London School of Hygiene & Tropical Medicine, United Kingdom

**Keywords:** Typhoid, Typhoid fever, Enteric fever, *Salmonella* Typhi, Vaccination, Immunisation, Transmission dynamics, Mathematical model, Economic evaluation, Cost utility analysis

## Abstract

Despite a recommendation by the World Health Organization (WHO) that typhoid vaccines be considered for the control of endemic disease and outbreaks, programmatic use remains limited. Transmission models and economic evaluation may be informative in decision making about vaccine programme introductions and their role alongside other control measures. A literature search found few typhoid transmission models or economic evaluations relative to analyses of other infectious diseases of similar or lower health burden.

Modelling suggests vaccines alone are unlikely to eliminate endemic disease in the short to medium term without measures to reduce transmission from asymptomatic carriage. The single identified data-fitted transmission model of typhoid vaccination suggests vaccines can reduce disease burden substantially when introduced programmatically but that indirect protection depends on the relative contribution of carriage to transmission in a given setting. This is an important source of epidemiological uncertainty, alongside the extent and nature of natural immunity.

Economic evaluations suggest that typhoid vaccination can be cost-saving to health services if incidence is extremely high and cost-effective in other high-incidence situations, when compared to WHO norms. Targeting vaccination to the highest incidence age-groups is likely to improve cost-effectiveness substantially. Economic perspective and vaccine costs substantially affect estimates, with disease incidence, case-fatality rates, and vaccine efficacy over time also important determinants of cost-effectiveness and sources of uncertainty. Static economic models may under-estimate benefits of typhoid vaccination by omitting indirect protection.

Typhoid fever transmission models currently require per-setting epidemiological parameterisation to inform their use in economic evaluation, which may limit their generalisability. We found no economic evaluation based on transmission dynamic modelling, and no economic evaluation of typhoid vaccination against interventions such as improvements in sanitation or hygiene.

## Highlights

• There are relatively few dynamic models or economic analyses of typhoid vaccination.
• The relative contribution of carriage to transmission is a key uncertainty.
• Published economic analyses use static models that omit indirect protection of vaccines.
• Nevertheless, vaccines appear highly cost-effective against WHO criteria in high-incidence settings.
• No economic model was found to compare vaccine and sanitation.

## 1 Introduction

Typhoid fever is an exclusively human enterically transmitted systemic disease caused by infection with the bacterium *Salmonella enterica enterica* serovar Typhi. Although largely controlled in Europe and North America, typhoid remains endemic in many parts of the world, notably Asia, where it is an important cause of febrile illness in crowded, low-income settings [1]. A notable feature of typhoid is the carrier state – asymptomatically infected individuals who continue to shed *Salmonella* Typhi in their stool or urine for many years, thereby sustaining transmission [2].

Despite a recommendation by the World Health Organization in 2008 that typhoid vaccination be considered for the control of endemic disease and outbreaks, programmatic use remains limited [3].

In the early twentieth-century, public health officials were debating the best methods of evaluating typhoid vaccine effectiveness, and whether vaccination was a distraction from improvements in sanitation and hygiene [4]. These remain contemporary policy issues for ministries of health and other health partners who may be considering programmatic anti-typhoid vaccination as a counterpart to other anti-typhoid measures such as improvements to income distributions, sanitation, water supplies and hand washing with soap (post-defecation and before the preparation of food in the home or sold in the street) as well as identification and management of carriage [5], [6], [7], [8]. Transmission dynamic modelling and economic evaluation are two informative tools to support such decisions [9], [10].

Where health budgets are limited, allocation of resources to activities which generate the best value for money maximises the population's health (not withstanding other health programme criteria such as equity). To compare between and across health states, cost utility analysis (CUA) can be employed using a common metric of health, such as disability-adjusted life-year (DALY). The World Health Organization's Choosing Interventions that are Cost-Effective project (WHO-CHOICE) describes interventions as “cost-effective” if they add a DALY at a cost of less than three times Gross Domestic Product (GDP) per capita, and “highly cost-effective” if each DALY costs less than GDP per capita. These are arbitrary thresholds and meeting them does not necessarily lead to the intervention being adopted, as health decision-makers are often required to make choices between multiple interventions that fall below these thresholds. Furthermore, even highly cost-effective activities may be too expensive overall for a health service to provide within budget: a hypothetical drug adding a year of life and costing GDP per capita for each person treated would require the entire national economy to be spent giving the drug to every member of the population [11].

By building on the germ theory of disease, and mass-action principles from the physical sciences [12], mechanistic mathematical modelling enables extrapolation beyond observed data, and can be used to project the expected trends of disease in a population or the potential impact of control strategies such as vaccination. Through capturing indirect effects of immunisation – the reduced incidence of disease in members of a population not themselves immunised, commonly described as “herd immunity” – these transmission dynamic models capture the impact of such interventions more completely than static economic models measuring only the direct effects in vaccinees [13].

In this review, typhoid transmission dynamic models and typhoid vaccine economic evaluations are examined for their potential contributions to informing disease control, identification of gaps in knowledge and indication of directions for further research.

## 2 Methods

PubMed was searched on 23 October 2014 without date restriction using the following terms: (“Typhoid Fever”[Mesh]) AND (“Nonlinear Dynamics”[Mesh] OR “Models, Theoretical”[Mesh] OR “Models, Statistical”[Mesh] OR “Computer Simulation”[Mesh] OR “Models, Economic”[Mesh] OR “Least-Squares Analysis”[Mesh] OR “Likelihood Functions”[Mesh] OR “Resource Allocation”[Mesh] OR “Cost-Benefit Analysis”[Mesh]) AND (Humans[Mesh]) NOT “Mice”[Mesh].

Personal libraries were reviewed and reference lists in papers searched for modelling and economic studies that may not have been identified by the above search strategy. Results were restricted to those available in English. We obtained information about unpublished studies through the Coalition against Typhoid and International Vaccine Institute.

Studies were included if they modelled typhoid transmission and/or analysed the cost-effectiveness of vaccination in endemic settings. Endemic settings were identified using recent high-quality reviews [14], [15]. We included cost of illness (COI) studies if they were linked to an analytical study, and willingness-to pay (WTP) studies if they included an economic evaluation or were linked to an analytical study. Studies were excluded if they used geographical or statistical modelling, including time-series analysis, without transmission dynamics, or if they addressed transmission or cost-effectiveness in non-endemic populations, such as international travellers.

Transmission models were assessed for their model structure, data sources, parameter estimates, use of fitting methods, sensitivity analysis and the contribution of their approach to epidemiological understanding of typhoid. Economic studies were evaluated by data sources, economic evaluation approach, perspective, comparator programmes, use of sensitivity analysis and capture of indirect effects of vaccination.

## 3 Results

Seventy-nine titles were retrieved. Ten modelling papers were selected for review based on title or abstract. One was discarded as a non-mechanistic time series study [16], one as it modelled outbreaks in a non-endemic setting [17], while two papers were of the same model and considered together [18], [19]. These are summarised in Tables 1 and 2. A further, as yet unpublished, transmission model has been developed by the International Vaccine Institute (personal communication, Jin Kyung Park) and is not reviewed here.

**Table 1:** Summary of typhoid transmission model types.

Characteristic	Number of models (*n* = 7)	References
Type of model		
Compartmental		
Deterministic	6	[18], [19], [20], [21], [22], [23], [24]
Stochastic	0	
Individual-based stochastic	1	[25]
Scope of model		
Analytical/mathematical		
Without data	1	[22]
Uses data without fitting	1	[23]
Exploratory/epidemiological		
Uses data without fitting	1	[21]
Fitted to data	1	[25]
Policy-oriented/public health		
Uses data without fitting	2	[18], [19], [20]
Fitted to data	1	[24]
Parameter-fitting method		
Maximum likelihood estimation	2 of 2	[24], [25]
Bayesian	0	
Investigates vaccination	4	[18], [19], [20], [21], [24]
Compares with improved sanitation, hygiene or water supply	4 of 4	[18], [19], [20], [21], [24]
Include economic evaluation of vaccination	2 of 4	[18], [19], [20]

Seven titles were identified as economic evaluation and obtained for full-text review (Table 3, Table 4) alongside two underpinning COI studies and one underpinning WTP study (Table 5). A further COI study was identified but excluded as not linked to a published economic evaluation [40].

**Table 3:** Summary of typhoid vaccine economic evaluation types.

Characteristic	Number of studies (*n* = 7)	Reference
Based on field studies	5	[27], [28], [29], [30], [31]
Perspective:		
Public sector only	2	[27], [32]
Private only	1	[29]
Societal (public and private)	4	[28], [29], [30], [31], [33]
Include intangible costs of pain, suffering and disability	3	[28], [30]
Analytical approach: (a study can include more than one approach)		
Cost-benefit analysis component	4	[29], [30], [31], [32]
Cost-effectiveness analysis component	2	[27], [32]
Cost-utility analysis component	2	[30], [33]
Willingness-to-pay component	4	[27], [28], [29]
Price-optimisation model	1	[27]
Include indirect protection of vaccines	1	[27]
Include transmission dynamics	0	
Evaluates improve sanitation, hygiene or water supply as an alternative to or adjunct to vaccination	0

**Table 4:** Components and main findings of typhoid vaccine economic evaluations.

	First author, year, reference	Analytical approach	Economic perspective	Setting	Burden of disease	Costs	Vaccine intervention modelled	Vaccine effectiveness
1	Musgrove 1992 [32]	CBA CEA	Public sector	PAHO SIREVA countries	150 cases per year per 100k population. CFR1% Does not cost pain, suffering or death.	Vaccine programs and clinical/field trials or pilots.	Mass vaccination; reducing number of doses over time.	Estimated 90%
2	Shepard 1995 [33]	CUA, cost per QALY	Public sector costs; societal benefit captured as QALYs	Countries with middle, high or very high U5MR	1.5 cases per person per lifetime. CFR 1.8% Morbidity is excluded from QALY estimates	Marginal costs of additional vaccination within a childhood programme	By birth cohort, two doses	Anticipated 80% over 10 y
3	Poulos 2004 [31]	CBA	Multi-dimensional public sector and societal	Kalkaji slum, New Delhi, India	As per [35]. Does not cost pain, suffering or death.	As above. Public funded vaccine programme.	Campaign with 80% coverage of: age 2–5, age 6–19, or all-age.	70% for 3 years
4	Canh 2006 [29]	WTP, contingent valuation, CBA	Private	Hue, Vietnam	Raised incidence 1995–9; associated with outbreak in 1996 Benefits measured by WTP.	Proposed USD 0.67 1.70 3.30 6.70 13.30	N/A	Proposed: 70%, 3 y; 70%, 20 y; 99%, 3 y; 99%, 20 y
5	Cook 2008 [30]	CUA	Public sector and societal	Kolkata, India; Karachi, Pakistan; North Jakarta, Indonesia; Hue, Vietnam	Highest in the sites within Karachi and Kolkata, lowest in Hue. Reported incidence double to account for false negative blood cultures. DALY weight 0.27, illness duration 7d CFR 1%.	Private direct and indirect cost of illness obtained in interviews with confirmed cases, public costs obtained from health facilities. Public and private vaccination costs from literature and estimation.	Campaigns: 1. School children 5 to 14 y 2. Children aged 2–15 y All 2 y+	65%, 3 y
6	Cook 2009 [28]	CBA total economic benefits vs costs 1. Societal COI 2. Above + Value of statistical life (VSL) saved 3. WTP (contingent valuation) + public costs	Societal	Tiljala and Narkeldanga slums, Kolkata, India	3.4 case per 1000 2–4 y 4.9 per 1000 5–15 y 1.2 per 1000 16 y+ DALY weight 0.27 CFR 1%	Total marginal vaccine cost USD (2007) $1.11 WTP as per [36] VSL from literature.	Campaigns: 1. School children 5–14 y 2. Children aged 2–15 y All 2 y+	65%, 3 y
7	Lauria 2009 [27]	Optimisation model: different adult and child pricing, implicit CEA	Public sector	Hypothetical population	3.5 annual cases per 1000 children and 1 per 1000 adults	As per [38]	Price-dependent uptake	70%, 3 y

	First author, year, reference	Time horizon	Discounting	Disease dynamics	Sensitivity analysis	Data source(s)	Findings
1	Musgrove 1992 [32]	14 and 24 years	10% pa	No	Program administration costs, vaccine costs, delay between accrual of costs and benefits	Expert opinion	Describes incidence, treatment costs and vaccination costs at which a program would be cost-neutral
2	Shepard 1995 [33]	10 years	3%, costs and benefits	No. Steady states pre-and post- vaccine program start. Assumes disease most common in late childhood or early adulthood.	1. Dose cost at USD50/QALY. 2. Vaccine development costs.	Expert opinion; extrapolation of high incidence epidemiological studies [34]	Preliminary estimate of highly CE (<USD50 per QALY, 1992 price) if data assumptions are valid. Critical parameters are incidence, CFR, VE, vaccine costs
3	Poulos 2004 [31]	3 years	10%	No	Incidence; vaccine cost; ratio of total economic benefit to measured COI	Bahl 2004 [35]	Immunisation of 2-5 year olds is cost saving to the public sector in a high incidence setting. Sensitivity analysis and inclusion of private costs suggest vaccination of other ages may also be highly CE.
4	Canh 2006 [29]	N/A	Inherent	Typhoid perceived to be in decline by 67% of participants	N/A	Cross sectional survey in 2002 of households with children	Survey participants are more sensitive to price than to expected vaccine efficacy or duration of protection. Modest user fees could support a vaccination programme.
5	Cook 2008 [30]	Over duration of vaccine	3%	No	Single parameters and Monte Carlo across all parameters, triangular distribution. VE 55% to 75%, duration 2–4 y,vaccine cost USD 0.40–0.80 (2007 prices), delivery cost variable. CFR 0.5–3%, illness duration 4 d to 3w, DALY weight 0.08–0.47.	DOMI	No programmes would be cost saving but (school-) child immunisation would be very CE to health services or society in all but Hue, including under sensitivity analysis. Adult vaccination in Kolkata and N. Jakarta is less CE but still meet thresholds. Surveillance likely reduced illness costs through early diagnosis.
6	Cook 2009 [28]	1 year cost, 3 year benefits	3%	No	As per [30]. VSL varied by 50%	Kolkata [37]	Economic perspective 1 is not cost neutral, but perspectives 2 and 3 indicate benefits greater than cost across all campaign strategies. Sensitivity analyses suggest WTP and VSL models show net benefit for all campaign strategies across most parameter sets.
7	Lauria 2009 [27]	3 y	8%	Possible herd protection described in a sensitivity analysis, with variable adult and child transmissibility.	Monte Carlo simulation, allowing most parameters to vary.	Five Asian countries [37]	There is minimal advantage to different vaccination charges for children and adults under the static model. Herd protection greatly influences case numbers and value.

CBA, cost-benefit analysis; CE, cost-effective(ness); CEA, cost-effectiveness analysis; CFR, case-fatality rate (proportion of cases that result in death); CUA, cost-utility analysis; COI, cost of illness; DALY, Disability adjusted life-year; DALY weight, a scale from 0 (perfect health) to 1 (death). DOMI, Diseases of the most impoverished programme [39]; PAHO, Pan-American Health Organization; SIREVA, Sistema Regional de Vacunas (Regional Vaccine System); U5MR, under-five mortality rate; USD, United States Dollars; VE, vaccine effectiveness; WTP, willingness to pay.

**Table 5:** Components and main findings of cost of illness studies and willingness to pay studies used in typhoid vaccine economic evaluations.

	First author, year, reference	Analytical approach	Economic perspective	Setting	Burden of disease	Costs	Vaccine intervention modelled	Vaccine effectiveness
1	Bahl 2004 [35]	Cost of illness	Multidimensional public sector and societal costs	Kalkaji slum, New Delhi, India	Culture confirmed incidence per year: 17 per 1000 under 5s; 12 per 1000 5–18 y; 1 per 1000 >19 y	Public sector/institutional and private costs, comprising direct medical, direct non-medical and indirect costs; for hospitalised and non-hospitalised	N/A	N/A
2	Poulos 2011 [38]	COI	Public and private (direct and indirect)	Hechi, China; North Jakarta, Indonesia; Kolkata, India; Karachi, Pakistan; Hue, Vietnam.	Highest in the sites within Karachi and Kolkata, lowest in Hechi and Hue.	Measured by questionnaire, with estimates for nonmarket activities. Karachi costs from expert information.	N/A	N/A
3	Whittington 2009 [36]	WTP	Private	Tiljala slum and Beliaghata neighbourhood, Kolkata, Inida	2 case per 1000 population per year, peak incidence in older children and teenagers	Proposed USD (2007) 0.22 0.56 1.11 11.11 And sliding scale.	Price-dependent uptake	70%, 3 y

	First author, year, reference	Time horizon	Discounting	Disease dynamics	Sensitivity analysis	Data source(s)	Findings
1	Bahl 2004 [35]	One year surveillance	N/A	No. Decline in incidence rate with age is informative of an immunising infection.	With both most conservative and least conservative cost estimates, and with incidence both on confirmed and clinically suspected disease.	Cohort study 1995–6, weekly interviews and passive surveillance.	Costs are high per episode regardless of age, both private and public/institutional. Hospitalisation and non-response to antimicrobials increase costs
2	Poulos 2011 [38]	N/A Interviews at 7, 14 and 90 d from disease onset.	N/A	N/A	N/A	Interviews with cases or carers.	Total episode costs range from USD 15–132. Private costs exceed public costs unless reimbursed. Hospitalisation adds to costs substantially. 14 to 49% of households borrowed money to pay for treatment. Costs of drug resistant infection are higher, but not significantly so.
3	Whittington 2009 [36]	N/A	Inherent	N/A	N/A	Cross sectional survey of households with children.	9% would decline a vaccine, with a further 7% only accepting free vaccine. WTP is at least USD2. Vaccines for children were valued higher than those for adults. Time to think reduces willingness to purchase vaccine.

There was minimal overlap found between transmission modelling and economic evaluation. Of the transmission dynamic models, only those by the Cvjetanović group also had cost-effectiveness components [18], [19], [20]. One economic study included quantitative consideration of indirect protection [27].

### 3.1 Transmission dynamic models

The seven typhoid models identified range from two-state analytical tools to complex individual-based simulation or multi-state compartmental models (see Table 1). Only two models were formally fitted to data [24], [25].

The structures of models (Table 2a, Table 2b) are based on different assessments or representations of the natural history of typhoid fever, particularly in how immunity to *Salmonella* Typhi is considered. González-Guzmán suggests that natural partial immunity is likely to arise but simplifies to a model with vaccine immunity only, noting that sufficiently high infectious doses can overcome immunity [21]. Pitzer uses population compartments to separately represent immunity against typhoid infection (‘sterile immunity’), and immunity against typhoid disease (‘clinical immunity’), allowing transition from the latter to either full susceptibility or to a subclinical infection that in turn restores full sterile immunity in the individual. This corresponds to immunity boosting repeated infection cycles without overt disease, particularly in adults after recovery from clinical disease in childhood, and allows bacterial shedding during subclinical infection to contribute to sustained transmission [24]. Saul similarly models both sterile and clinical immunity, with infection resulting in sterile immunity that wanes to clinical immunity (potentially after zero time) and then to susceptibility, and explores a range of hypothetical state-transition scenarios based on multiple infections, though he does not clearly resolve a most-likely scenario [25]. Despite asymptomatic boosting being a long-standing hypothesis, or perhaps because of it, there is a paucity of data from microbiological, immunological or epidemiological studies to parameterise models or to validate assumptions [41].

**Table 2a:** components and main findings of typhoid transmission models.

	First author and year		Model type	Disease states	Data source(s)	Fitting process	Interventions modelled	Time horizon	Sensitivity analysis	Findings	Comments
1	Cvjetanović 1971, 1978	[18][19]	Compartmental deterministic with births = deaths, without age-structure	N S E_s_ E_a_ I_s_ I_a_ C_t_ C_l_ R_t_ R_l_	Parameters estimated using literature and expert opinion. Considers an eidemiological scenario approximating Western Samoa.	None	Vaccination with whole-cell inactivated vaccines, VE 60%, 75% or 90%, coverage 60, 80 or 100%. As one-off or 5 yearly campaigns. Sanitation	60 years	Epidemiological/clinical parameters fixed. Effective contact rate (per capita per day) varied.	For both low and high VE, single vaccination campaigns achieve temporary reduction in incidence rates before return to a rate determined by the force of infection, where force of infection is above an elimination threshold. Sustained reduction in force of infection reduces incidence. Multiple vaccination campaigns reduce incidence will campaigns are sustained.	Multiple parameters are included without fitting. Outputs should be considered illustrative.
2	Briscoe 1980	[22]	Deterministic analytical SIS	S I	N/A	N/A. Reviews Cvjetanović models. Analysis of role of force of infection and recovery on equilibrium prevalence.	N/A	N/A	N/A	Force of infection determines prevalence, and vice versa. Stochasticity may prevent disease eradication.	Intended as an analytical model rather than epidemiological simulation.
3	Bailey 1982	[23]	Compartmental deterministic with births = deaths, without age-structure	S E I C R	[18]	Rule-based simplification of Cvjetanović 1971 model [18] with direct mathematical solution of steady-state equations.	N/A	N/A	N/A, suggests an approach to sensitivity analysis [26]	For a steady-state model, structural simplification results in compartment population estimates consistent with the unsimplified model for a given effective contact rate.	Reducing the number of compartments makes a model more suitable for validation with data.
4	Cvjetanović 1986	[20]	Age-structured compartmental deterministic SIRS. Birth and death rates from Chile	N S I C_t_ C_l_ R_t_ R_l_	Demographic and typhoid surveillance data for Santiago and rest of Chile	Effective contact rate per capita per unit day (age-specific for acquisition) from linear interpolation of age-specific incidence. Visual goodness-of-fit. Strong assumption that 20% of all cases are clinical.	Vaccination with Ty21a, 95% VE at 75% or 95% coverage of under 25s with 5 yrly revaccination. Food sanitation in schools reducing force of infection by 1/3 in ages 6 to 16 y. Sanitation with annual 2% or 5% improvement in force of infection over 10 years.	Interventions analysed over 25 y after run-in to equilibrium.	None	Vaccination campaigns would reduce age-specific incidence and increase the age of peak incidence Vaccination would not eliminate disease over 25 y but would result in year on year reduction in incidence if sustained. 10 y sanitation campaigns likely to reduce prevalence and continue to reduce prevalence after cessation.	Somewhat simplified model structure, though now age structured. The model is not validated sufficiently against data, nor are outputs sufficiently clear to make strong policy conclusions. Age-based changes in incidence with vaccination are consistent with epidemic theory.
5	González-Guzmán 1989	[21]	Compartmental deterministic SIS structure with births and deaths	S I V with environmental transmission	Parameter estimates for Chile	None, analytical model	Reductions in combinations of: carrier prevalence indirect contact rate direct contact rate environmental life of the bacterium bacterial count in the environment. Vaccination with Ty21a, coverage scaled for equivalence to VE 74% or 95%.	10 y	N/A	Decline in incidence is not rapid, even with highly effective combined control measures. Reduction in chronic carriage most effective control procedure. Vaccination as a permanent programme would require a high proportion of the population to become immune to control typhoid within a meaningful timeframe.	Author cautions against using the model to estimate the effect of a vaccination programme but that it indicates areas for further epidemiological parameter determination.
6	Saul 2013	[25]	Individual-based stochastic, random-mixing.	S E I_s_ I_a_ C_t_ C_l_ R_t_ R_l_, R_c_,;R_s;_ V_c_ V_s_	Surveillance data from Dhaka, Bangladesh, and Kolkata India. Migration, birth and death rates from Matlab, Bangladesh. Other parameter from literature and expert opinion.	Maximum likelihood and visual inspection	None	40 y to equilibrium and 40 y follow-up. 20 y for effects of carriage.	Sensitivity analysis on refractory period from birth.	Distinguishes between sterile immunity and clinical Immunity (in which individuals can be infected but not develop disease). Multiple infections needed to develop sterile immunity. Natural immunity is likely to be long-lasting but needs further field investigation. Carriage stabilises dynamics, and is particularly important in lower incidence settings.	Complex agent based model, limited availability of epidemiological data results in issues of parameter identifiability. Plausible combinations of parameters identified.
7	Pitzer 2014	[24]	Compartmental, age-structured deterministic	S_1_ S_2_ I_1_ I_2_ R C W	Surveillance case series, Vellore, Tamil Nadu, India	Two-stage fitting with Latin hypercube sampling of starting parameters. Maximum likelihood estimation, simplex method.	Vaccination with: Ty21a, (VE 48%, duration = natural immunity), Vi polysaccharide (VE 80%, 3 y), Vi conjugate (VE 95.6%, 19.2 y). Vaccination of school age children as a campaign, routine vaccination of 6 year olds, or both together. Improvements in water and sanitation over 30 y	50 y to quasi-steady state and 25 y follow-up	Multi-parameter sensitivity analysis in model fitting.	Basic reproduction number is around 3 in Vellore and 7 in Dhaka. Natural immunity is likely to be long-lasting. Vaccination campaigns would not eliminate disease in Vellore but instead see disease rebound in 5 to 10 y. A campaign plus routine immunisation could result in a new lower incidence disease state. High baseline carriage rates reduce the indirect protection of vaccines–understanding carriage prevalence should be a disease control priority. In most circumstances modelled, improvements in hygiene and sanitation have more impact than vaccination.	Best fitting parameter sets were highly sensitive to initial parameter selection. Identifies carrier transmissibility and relative contributions of short- and long- cycle as import epidemiological sources of uncertainty.

VE = vaccine efficacy. Effective contact rate is the rate at which two individuals come into contact per unit time, with the nature of the contact being such that if one was infectious and the other susceptible, infection would be transmitted.

**Table 2b:** Disease states in typhoid models.

Abbeviation	Disease state	Comment
N	Newborn	Susceptible in Cvjetanović’s model, refractory in Saul's
S	Susceptible;	
S_1_ S_2_	Fully and partially susceptible	
E;	‘Exposed’;	Infected but not (yet) infectious
E_s_; E_a_	Symptomatic or asymptomatic	
I;	Infectious;	
I_s_ I_a_	Symptomatic or asymptomatic	Primary infection of a fully susceptible individual or asymptomatic/subclinical infection of a previously partially susceptible individual
I_1_ I_2_	Primary or subclinical infection	
C;	Carrier;	
C_t_; C_l_	Temporary; long-term	
R;	Removed/resistant/refractory/recovered;	Not able to be infected, immune.
R_t_; R_l_;	Temporary immunity; long-term immunity;	Clinical immunity is against disease but allows infection and onward transmission.
R_c_; R_s_	Natural immunity to clinical disease; natural sterile immunity)	Sterile immunity is against any infection.
V	Vaccinated	
V_c_; V_s_	Vaccine-induced immunity to clinical disease; Vaccine-induced sterile immunity	
W	‘Water’	Long-cycle transmission from water or environmental contamination, contributed to by all infectious or carrier classes.

While noting leaky immunity in those naturally infected (each individual has a reduced probability of further infection), González-Guzmán models Ty21a oral vaccine protection as all-or-none, giving each vaccinee a probability of developing immunity or not. In this model, those who develop immunity following vaccination have 100% protection against typhoid, until vaccine wanes and they return to full susceptibility, an approach also applied by Cvjetanović [18], [19], [20], [21]. Pitzer handles injected Vi vaccination the same, noting results were similar in a sensitivity analysis assuming leaky vaccine immunity. Pitzer represents oral Ty21a vaccination as akin to natural immunity, transitioning vaccinees to clinical immunity after full immunity wanes [24].

While vaccination programmes are predicted to reduce typhoid incidence, uncertainty around carriage prevalence, duration and contribution to the force of infection substantially affects vaccines’ projected impact [21], [24], [25]. In reviewing Cvjetanović’s 1978 model [19], Anderson and May observe that the implicit assumption that the effective contact rate for carriers is equal to that of acute cases, combined with other fixed parameter estimates, gives carriers a contribution to transmission ten times that of other cases [42]. While illustrative of the potential contribution of carriage in sustaining disease, for policymaking it has been recommended that such assumptions should be tested against data [43], [44]. An approach might be to conduct systematic, detailed investigation of incidence cases to identify potential sources, using suitable screening methods to look for carriers as well as acute cases, and combine this with population-level carriage surveys and water quality studies. While labour-intensive, such investigations could be integrated into wider control efforts [45].

Chronic *Salmonella* Typhi carriage can be treated with antibiotics and/or cholecystectomy for gallstone-associated infection, but there is no demonstrated role for vaccination in clearance of carriage [46], [47]. Premised on this, Cvjetanović’s and Pitzer's models demonstrate that where carriage contributes substantially to transmission in endemic settings, vaccination is unlikely to result in elimination in the short-to-medium term [18], [24]. Similarly, where carriage rates are high the indirect protective effects of vaccination are reduced, as the risk to the unvaccinated of acquiring disease from carriers is not diminished [24], [42]. The contribution of carriage, however, requires further epidemiological assessment, as does the role of short-cycle and long-cycle (environmental) transmission [21], [24], [25].

If immunisation of the susceptible population does not bring about typhoid elimination, then measures to reduce per case or per carrier infectivity, such as improved sanitation or hand washing with soap, might be considered instead of or in conjunction with vaccination [48], [49]. The multi-compartment models suggest such a reduction in effective contact rates could lead to important reduction in prevalence [21], [23], [24]. This is consistent with Briscoe's analytical model [22].

Another feature of transmission dynamic studies is that the average age of infection increases as the force of infection decreases, for example, with the introduction of vaccine [20]. This is consistent with burden of disease studies which find earlier average age of infection in settings with higher disease incidence [14], [35].

### 3.2 Economic evaluation

Our literature search found seven papers evaluating typhoid vaccine cost effectiveness. The earliest two of which were based on values derived from expert opinion and are less informative to current policy considerations than the most recent five which were based on field studies, as outlined in Table 3, Table 4. Two supporting COI studies and one supporting WTP study are outlined in Table 5. These field-informed analyses share multiple common authorships with collaboration through the Diseases of the Most Impoverished (DOMI) programme. Of the seven economic evaluations, four included a cost-benefit analysis (CBA), two a cost-effectiveness analysis (CEA) and two a cost-utility analysis (CUA). Four of these used a societal perspective [28], [30], [31], [33]. Only one evaluation considers indirect protection quantitatively, but uses hypothetical values for herd immunity from different coverage levels rather than estimates from dynamical modelling [27]. No studies considered improvements in sanitation, hygiene or water supply as an alternative or adjunct to typhoid vaccination.

In a one-year study of a very-high typhoid incidence area – Kalkaji slum, Delhi – Bahl found average costs per episode of illness were high to both the health sector and families, excluding intangible costs such as pain, with hospitalisation an important component of health service costs [35]. A CBA by Poulos on this data reported that a vaccination programme for children under five years of age would be cost-saving to the public sector. Analysis from a societal perspective, incorporating private costs, indicated that vaccination in high incidence settings with modestly priced vaccines could also have net benefits in other age groups [31].

Cook and colleagues conducted a CUA based on field data from multiple Asian sites [30], [38], and found that while typhoid vaccination using the Vi-polysaccharide across adults and children would be unlikely to be cost saving to the public sector, in high incidence settings it was likely such a programme would meet the standard for “very cost-effective” health interventions of less than per-capita gross domestic product (GDP) per DALY gained. In these settings, targeting vaccination to the highest incidence age-groups improved cost-effectiveness substantially. Through sensitivity analysis, the main determinants of cost-effectiveness identified were vaccine cost, case-fatality rates (CFR), vaccine duration of protection, (baseline) incidence and vaccine efficacy. Cost-effectiveness was insensitive to vaccine coverage as no indirect protection was assumed [30].

WTP has also been used in economic evaluation of typhoid vaccines as an alternative approach to COI in valuing private or societal benefits. Such an approach is considered to demonstrate the value individuals place on the total benefit of the vaccine, though is confounded by ability to pay, and ability to value public sector activities foregone if vaccines are supplied through the state [50]. One study from Hue, Vietnam suggested that typhoid vaccination would pass a social cost-benefit test (total costs less than total societal benefit), based on demand estimates from a contingent valuation survey addressing hypothetical vaccine purchases for householders and their children [29]. Analysis by Cook of a similar WTP study done in Kolkata, India, by Whittington and colleagues, suggested that vaccination of children or all-ages would not pass a social cost-benefit test using COI, but costing benefits using WTP plus public costs would likely find that such programmes pass such a test [28], [36].

WTP studies are also informative to vaccine uptake, with 9% of respondents in the Kolkata survey stating they would not accept a free vaccine, with data suggesting these individuals are more likely to be older, have lower income and never boil drinking water [36]. In the Hue survey, Canh suggests a number of issues affect validity of economic evaluation using WTP estimates, noting that householders were most sensitive to price, with proposed vaccine efficacy making no detectable difference to individual or household demand at a given price [29].

Observing that typhoid vaccines are equivalent to one-sixth of per-capita public sector health spending in India, Cook notes the potential for user fees in financing a state-administered programme [28]. Whittington's Kolkata WTP survey suggested vaccine protection for children was given greater value than vaccinating adults [36]. Drawing on data from this study, a price optimisation model by Lauria of different vaccine prices for children and adults did not find a strong case for differential pricing, but in a non-dynamic sensitivity analysis of potential indirect protection scenarios found herd immunity to be a significant influence on incidence and cost-effectiveness [27].

## 4 Discussion

This review found a relatively sparse literature on typhoid modelling and vaccine economic evaluations. Of seven transmission models found, only two were published in the last 25 years and use contemporary data-fitting methods. All five field-based economic evaluations we found shared multiple common authorships around the DOMI collaboration.

Although the Global Burden of Disease is a much-criticised ranking, it provides some comparator infectious diseases, with measles and syphilis ranked close to typhoid and paratyphoid fever, and cholera attributed around one-third of the annual number of DALYs [51]. Repeating our (non-comprehensive) PubMed typhoid search strategy for these returned over six times as many measles papers, three times as many syphilis papers, and twice as many cholera papers. Typhoid fever transmission and economic evaluation appear relatively under-studied. Typhoid's low profile-to-burden ratio has variously been attributed to ubiquity in developing countries, inadequate diagnostic tools, the absence of champions in health agencies, and affecting mostly the poor and the underclasses [52]. Effective antibiotics and previous non-availability of a long-acting infant vaccine have also been cited as reasons for the absence of internationally funded typhoid vaccination programmes [52]. For budget-constrained national health agencies, typhoid vaccination programmes may appear unattractive unless public sector cost saving can be demonstrated, for example, if the incidence rate is very high [31], [35].

Considering only a health services perspective – of treating typhoid and providing vaccine – omits private costs associated with the disease and therefore underestimates the societal impact of typhoid. Various approaches have been taken to address these costs and more completely capture the benefit of vaccination, from cost of illness studies for private expenses, estimates of health utility forgone due to illness, or by determining the extent to which people value vaccination in willingness-to-pay studies. While user fees and WTP are controversial [10], [50], economic evaluations that consider these have been consistent with typhoid economic analysis using more widely accepted cost-utility analysis and private cost of illness. These CUAs suggest typhoid vaccine programmes are likely to be highly cost-effective (against international norms) where disease is highly endemic, particularly when targeted to the age-groups at highest risk of disease. The economic analyses, which do not include mechanistic components for transmission, emphasise as key drivers of cost-effectiveness the vaccine cost, case fatality rates, baseline incidence, vaccine efficacy and duration of protection.

We did not find any economic evaluation comparing typhoid vaccination programmes against other potential means of typhoid control, such as measures to improve sanitation, hygiene or water supplies. It is still not possible to answer the century-old question of whether, in a given setting and within a limited budget, vaccination should be adopted over improved sanitation and hygiene, or what combinations are optimal for control under what circumstances. Such analysis would need to be based on a transmission dynamic model, with extensive epidemiological surveillance for detailed burden of illness measurement, and comprehensive costing for CUA or CBA approaches that allow other diseases to be included in the evaluation.

The only published data-fitted transmission model of typhoid vaccination suggests that while vaccination is effective in reducing disease incidence, if other measures are not enacted to reduce the ongoing force of infection, particularly from asymptomatic carriage, short or medium-term vaccination campaigns are unlikely to result in elimination and would see disease rebound if vaccination stopped [24]. The authors of recent dynamic models emphasise our lack of understanding of certain aspects of the natural history of typhoid (particularly around acquisition of immunity, the role of carriers, and the contribution of short- and long-transmission cycles) [21], [24], [25]. A model intended to examine what role the putative different forms of *Salmonella* Typhi immunity have in determining typhoid incidence rates found an absence of suitable immuno-epidemiological data on which to fit parameters and make strong inferences, a challenge further compounded by the absence of age structure in the model [25].

Transmission dynamic modelling and a non-mechanistic economic analysis have shown that the level of indirect protection may have an important impact on vaccine effectiveness and cost-effectiveness respectively [24], [27]. None of the economic models mechanistically consider disease dynamics and so cannot scientifically appraise the indirect effects of vaccination in cost-effectiveness calculations. Indeed, while a number of economic analyses readily acknowledged indirect effects as an important phenomenon, they specifically excluded them, citing the absence of evidence for Vi polysaccharide vaccine herd immunity pending the publication of cluster randomised controlled trials [28], [30], [53], [54]. Early work by Cvjetanović is the only meeting point we found of mechanistic typhoid transmission modelling and economic analysis, but the complexity of this model and absence of fitting make it difficult to apply findings to contemporary disease control problems [18], [19].

Using a static economic model premised only on direct protection in vaccinees may be a reasonable approximation in some situations, such as if vaccine-preventable new typhoid cases (symptomatic or otherwise) make a relatively small contribution to the force of infection compared with carriers and the unimmunised. Even in such circumstances, prior assessment with mechanistic modelling of field epidemiological data would be appropriate in estimating the relative contributions of each group to transmission.

While there seems limited inter-disciplinary dialogue between typhoid modellers and economists, a unifying concern is the importance of accurately determining age-based incidence rates, which can be highly variable within-country or between otherwise similar settings, and are central to estimates of vaccine impact and cost-effectiveness. Heterogeneity in disease rates, transmission mechanisms and health service provision may limit external validity of both typhoid modelling studies and economic evaluations.

Accurate assessment of disease burden could be done with large, population-based studies to inform incidence, complications and case-fatality rates, using blood culture confirmation of cases, or altogether improved diagnostics [30], [55], [56], [57]. It should be noted that even well-conducted studies are unlikely to provide unbiased estimates, due to the positive health consequences of introducing disease surveillance. Bahl notes that active surveillance with early treatment gave rise to disease that is less severe, and less expensive, than disease detected through passive surveillance [35]. This is echoed in the DOMI disease burden study, which rather than the 1% case-fatality rate widely cited in literature, had a zero percent CFR amongst the 475 cases detected (which gives an upper 95% confidence interval of around 0.63%) [30], [37], [58].

Other field epidemiology and laboratory investigations could further inform typhoid transmission dynamics [59]:

• Large, population-based, *Salmonella* Typhi carriage studies, similar to those done in Chile in the early 1980s [5], to determine prevalence in a range of endemic settings, potentially with serological surveys [60], alongside investigation of potential sources of infection amongst new cases.
• Serological assay development and population-based serological surveys to determine past infection to *Salmonella* Typhi, natural immunity and waning of this immunity. Seroprevalence could be linked to surveillance records to estimate the proportion of infections that are clinically apparent and notified to national authorities.
• Epidemiological time series with consistent, transparent methodology and/or cross-referencing between methods [61].
• Age-based social contact pattern surveys, which may inform short-cycle transmission [62].

For models to assess vaccination against other enteric fever control measures, findings could be incorporated from interventional field studies on the role of improvements to sanitation, hygiene and water supply in changing disease incidence and transmission. Ongoing scrutiny of vaccine efficacy and duration of vaccine protection may also be informative. Estimates of efficacy of Ty21a vaccines in recent systematic reviews are less than in the data sources for early modelling, with the reviews focusing on individual RCTs rather than cluster field studies [20], [63], [64], [65], [66]. Estimates for Vi-polysaccharide effectiveness have also been modified downward [31], [64]. Analysis of differences between cluster and individual randomised trials may be informative on indirect protection.

This review has a number of limitations. In the absence of licensed human vaccines, the review does not cover paratyphoid fever or non-typhoidal salmonelloses. It covers only material in the English language, limited searches to a non-systematic enquiry of a single database and does not attempt to synthesis qualitatively or quantitatively any of the studies reviewed. Comparator studies have not been sought that consider investments in water, sanitation and hygiene as alternatives to typhoid vaccination.

One possible feature observed in the course of this review is a less pessimistic assessment of disease burden, perhaps reflecting true decline, as well as a more sceptical perspective on vaccine efficacy estimates, with fewer inputs based on expert opinion alone. Any such trend towards assessment of vaccine costs and benefit firmly grounded in data is beneficial to equitable, scientifically informed health-policy setting.

It has been suggested that for a model to have sufficient complexity to enable robust cost-effectiveness analysis, substantial data collection may be required [67]. When data is in short supply, theoretically informed modelling may still be a particularly appropriate tool to support decision-making [68], [69]. Transmission modelling using existing data explains patterns seen in average age of infection, demonstrates the importance of carriage, suggests optimal strategies for vaccination, and appraises the potential role of other interventions to reduce transmissibility.

## 5 Conclusion

Transmission dynamics have not yet been integrated into a comprehensive cost-utility analysis of typhoid vaccination and, as such, there is no economic evaluation that would meet contemporary gold standards [9], [70]. Given the costs and time involved in further field study, constructive efforts could be made to integrate existing transmission modelling and cost-effectiveness analyses, such as utilising the extensive collation of typhoid epidemiological and clinical parameters by Saul [25] with the transparent, reproducible modelling approach of Pitzer [24], and DOMI project economic data [30], [38]. While such endeavours would not address the fundamental limitations on health service budget in endemic areas, an analysis of typhoid vaccination that enables economic comparison across health arenas could help bring into the public gaze the full potential of measures to control enteric fever, and improve the prospects of protection from typhoid for people living with daily risks from a disease eliminated from most of the affluent world.

## Ethics

Literature review, ethics approval not required.

## Conflict of interest

CHW and WJE have had travel and expenses paid for by the Coalition against Typhoid to attend meetings on the modelling of typhoid vaccination programmes. WJE has undertaken consultancy for the Coalition against Typhoid, which was paid to a fund held the London School of Hygiene & Tropical Medicine.

## Authors’ contributions

CHW conducted the review and interpretation, wrote the initial draft of the manuscript and revisions. WJE contributed to the interpretation, and reviewed and edited the draft manuscript.
